# Production of 3-hydroxypropionic acid in engineered *Methylobacterium extorquens* AM1 and its reassimilation through a reductive route

**DOI:** 10.1186/s12934-017-0798-2

**Published:** 2017-10-30

**Authors:** Yi-Ming Yang, Wen-Jing Chen, Jing Yang, Yuan-Ming Zhou, Bo Hu, Min Zhang, Li-Ping Zhu, Guang-Yuan Wang, Song Yang

**Affiliations:** 1 School of Life Science, Qingdao Agricultural University, Shandong Province Key Laboratory of Applied Mycology, and Qingdao International Center on Microbes Utilizing Biogas, Qingdao, Shandong Province China; 2 Central Laboratory, Qingdao Agricultural University, Qingdao, Shandong Province China; 3 Department of Chemical Engineering, University of Washington, Seattle, WA USA; 4 Key Laboratory of Systems Bioengineering, Ministry of Education, Tianjin University, Tianjin, China; 5 Present Address: Industrial Product Division, Intrexon Corporation, South San Francisco, CA 94080 USA

**Keywords:** *Methylobacterium extorquens*, Methanol, 3-Hydroxypropionic acid, Reassimilation, Reductive route, ^13^C-labeling

## Abstract

**Background:**

3-Hydroxypropionic acid (3-HP) is an important platform chemical, serving as a precursor for a wide range of industrial applications such as the production of acrylic acid and 1,3-propanediol. Although *Escherichia coli* or *Saccharomyces cerevisiae* are the primary industrial microbes for the production of 3-HP, alternative engineered hosts have the potential to generate 3-HP from other carbon feedstocks. *Methylobacterium extorquens* AM1, a facultative methylotrophic α-proteobacterium, is a model system for assessing the possibility of generating 3-HP from one-carbon feedstock methanol.

**Results:**

Here we constructed a malonyl-CoA pathway by heterologously overexpressing the *mcr* gene to convert methanol into 3-HP in *M. extorquens* AM1. The engineered strains demonstrated 3-HP production with initial titer of 6.8 mg/l in shake flask cultivation, which was further improved to 69.8 mg/l by increasing the strength of promoter and *mcr* gene copy number. In vivo metabolic analysis showed a significant decrease of the acetyl-CoA pool size in the strain with the highest 3-HP titer, suggesting the supply of acetyl-CoA is a potential bottleneck for further improvement. Notably, 3-HP was rapidly degraded after the transition from exponential phase to stationary phase. Metabolomics analysis showed the accumulation of intracellular 3-hydroxypropionyl-CoA at stationary phase with the addition of 3-HP into the cultured medium, indicating 3-HP was first converted to its CoA derivatives. In vitro enzymatic assay and β-alanine pathway dependent ^13^C-labeling further demonstrated that a reductive route sequentially converted 3-HP-CoA to acrylyl-CoA and propionyl-CoA, with the latter being reassimilated into the ethylmalonyl-CoA pathway. The deletion of the gene META1_4251 encoding a putative acrylyl-CoA reductase led to reduced degradation rate of 3-HP in late stationary phase.

**Conclusions:**

We demonstrated the feasibility of constructing the malonyl-CoA pathway in *M. extorquens* AM1 to generate 3-HP. Furthermore, we showed that a reductive route coupled with the ethylmalonyl-CoA pathway was the major channel responsible for degradation of the 3-HP during the growth transition. Engineered *M. extorquens* AM1 represents a good platform for 3-HP production from methanol.

**Electronic supplementary material:**

The online version of this article (10.1186/s12934-017-0798-2) contains supplementary material, which is available to authorized users.

## Background

3-Hydroxypropionic acid (3-HP) is an important platform chemical, serving as a precursor for a wide range of industrial applications such as the production of acrylic acid and 1,3-propanediol [1, 2]. Additionally, 3-HP can be polymerized alone to generate poly-3-hydroxypropionate or in combination with other monomers to obtain biodegradable polymers [3, 4]. There are two major routes that have been developed for 3-HP synthesis in engineered microorganisms including *Escherichia coli*, *Saccharomyces cerevisiae* and *Pyrococcus furiosus*. One route is referred as the malonyl-CoA pathway, which reduces malonyl-CoA to synthesize 3-HP by either a bifunctional malonyl-CoA reductase (Mcr) or the combined malonyl-CoA reductase and malonate semialdehyde reductase [5–8]. Another recently developed route is called the β-alanine dependent pathway, which involves decarboxylation of aspartate to β-alanine and conversion of β-alanine to malonate semialdehyde then 3-HP [9, 10]. So far the highest titer of 3-HP production (3.7 g/l) by expressing the malonyl-CoA pathway in shake flasks has been achieved in *E. coli* grown on glucose [11, 12]. It is noteworthy that in nature 3-HP serves in central metabolism either as an intermediate of C3/C4 assimilation cycles or as an input carbon source in some bacteria and archaea. For example, *Chloroflexus aurantiacus* can utilize 3-HP as an intermediate in the 3-HP/malyl-CoA cycle to assist in two CO_2_ fixation pathways [13], and *Rhodobacter sphaeroides* can utilize 3-HP as the sole carbon and energy source by reducing 3-HP to 3-hydroxypropionyl-CoA, then through acrylyl-CoA to propionyl-CoA for incorporating into cell components [14]. Furthermore, in some engineered bacteria such as *Pseudomonas denitrificans*, 3-HP is rapidly degraded and reassimilated in the late period of cultivation through an oxidative route leading to the generation of acetyl-CoA and CO_2_ [15–18]. Hence, understanding the function and regulation of reassimilation is another important issue for stably enhancing the titer of 3-HP.

One goal of metabolic engineering is to develop renewable and sustainable alternatives to produce 3-HP. In particular, methanol is an important C1 feedstock that can be generated from synthesis gas (a mixture of CO and H_2_) or from biogas, assuming high quantity and low market price [19, 20]. *Methylobacterium extorquens* AM1, a facultative methylotrophic α-proteobacterium capable of using methanol and multiple carbon compounds as carbon and energy sources, has served as the best-characterized model organism for studying one-carbon metabolism [21, 22]. In *M. extorquens* AM1 assimilation during methylotrophic metabolism involves three interlocked cycles: the serine cycle, the ethylmalonyl-CoA pathway (EMC pathway) and the poly-3-hydroxybutyrate (PHB) cycle [23, 24]. The main function of the EMC pathway is to regenerate glyoxylate from acetyl-CoA for reincorporation into the serine cycle during C1 assimilation [25–29]. It has been shown that significant metabolic flux goes through the serine cycle and EMC pathway during the cell grown on methanol, generating a stable supply of acetyl-CoA as a precursor for the production of mevalonate acid, α-humulene and 1-butanol in engineered *M. extorquens* AM1 [30–33]. This feature is an advantage for engineering a modified malonyl-CoA pathway in *M. extorquens* to produce 3-HP as acetyl-CoA can be directly converted to malonyl-CoA. In addition, the growth rate of *M. extorquens* AM1 on methanol is significantly faster than that of *C. aurantiacus* which has native pathway to produce 3-HP from CO_2_ fixation [34], which added another advantage to use *M. extorquens* AM1 as a C1 platform for 3-HP production.

When *M. extorquens* AM1 is grown on multicarbon compounds, they are utilized through different entry points. Succinate has an entry point at the tricarboxylic acid cycle (TCA cycle), and pyruvate can be utilized through an overlap of the serine cycle and TCA cycle [35, 36]. Acetate is first converted to acetyl-CoA, which is further assimilated through both the EMC pathway and TCA cycle [37]. Moreover, some dicarboxylic acids such as methylsuccinic acid and mesaconic acid are also able to be assimilated via the EMC pathway although the growth rate is much slower than that on succinate and methanol [38]. 3-HP is a molecule of carboxylic acid and propionyl-CoA involved in its natural assimilation is an intermediate of the EMC pathway as well. From the viewpoint of carboxylic acid use in *M. extorquens* AM1, it is important not only to study how to improve 3-HP titer but also to investigate whether there is a loss of 3-HP due to product reuptake.

**Fig. 1:**
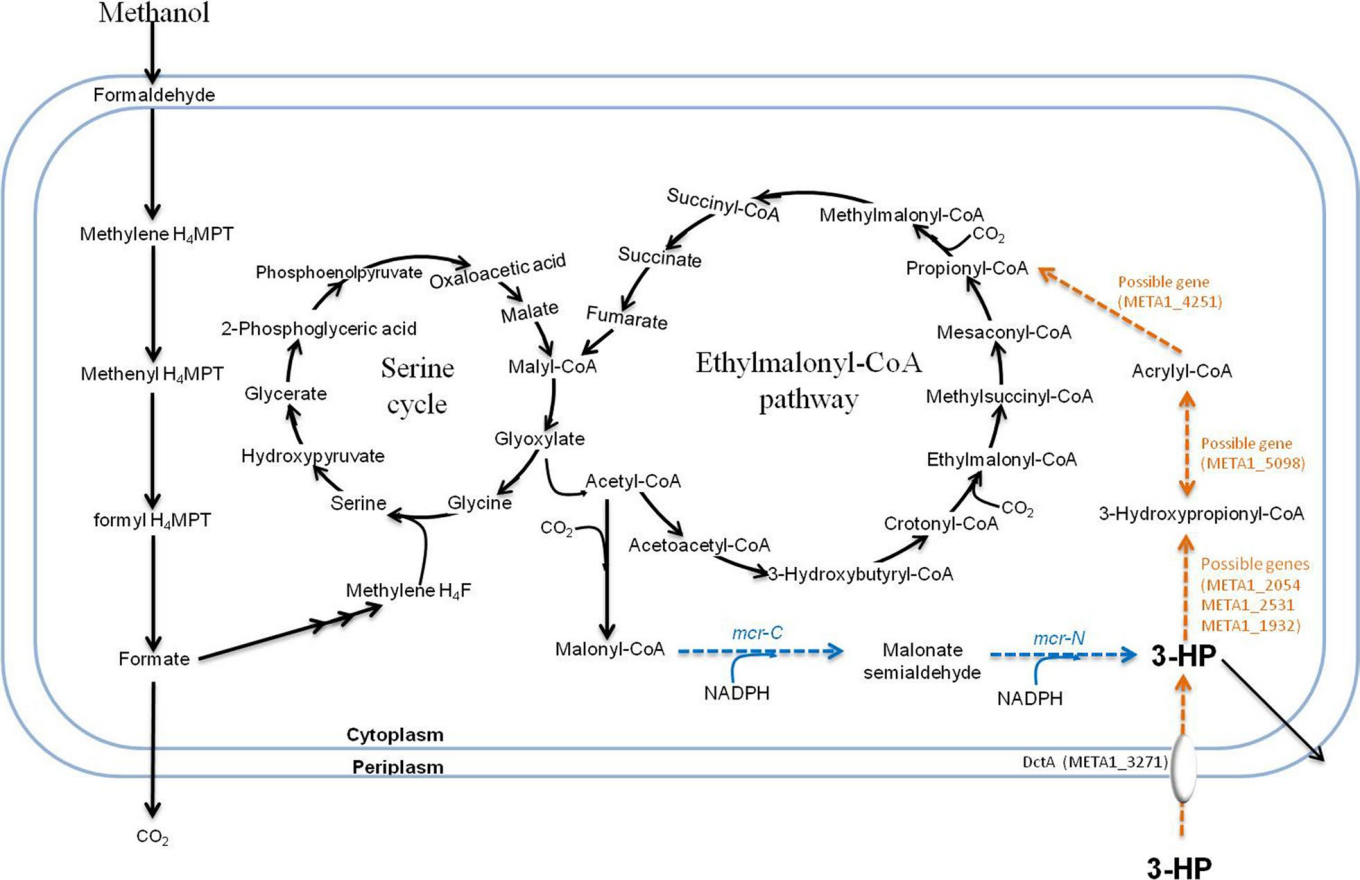
Constructing the heterologous malonyl-CoA pathway in *M. extorquens* AM1 for 3-HP production. Mcr-N and Mcr-C: N- and C-terminal fragments of malonyl-CoA reductase. Black solid lines represent methylotrophic pathways used for methanol assimilation. Blue dashed lines represent the malonyl-CoA pathway for 3-HP production. Orange dashed lines represent the 3-HP reassimilation pathway, and possible genes involved in reassimilation were added accordingly

In this study, we constructed and tuned the malonyl-CoA pathway to produce 3-HP (Fig. 1) and then improve 3-HP titer in *M. extorquens* AM1 grown on methanol. Further, we used metabolomics, enzymatic assay and β-alanine pathway dependent ^13^C-labeling as a combined approach to demonstrate a reductive route coupled with the EMC pathway as the major route for reassimilation of 3-HP into central metabolism upon the transition from exponential phase to stationary phase. We also determined how the deletion of an acid transporter and putative acrylyl-CoA reductase from *M. extorquens* AM1 affected 3-HP degradation. This research sheds light on engineering *M. extorquens* AM1 as an alternative microbial factory for producing 3-HP and related downstream products such as acrylic acid from methanol.

## Results

### Construction of a malonyl-CoA pathway for 3-HP production in *M. extorquens* AM1

**Fig. 2:**
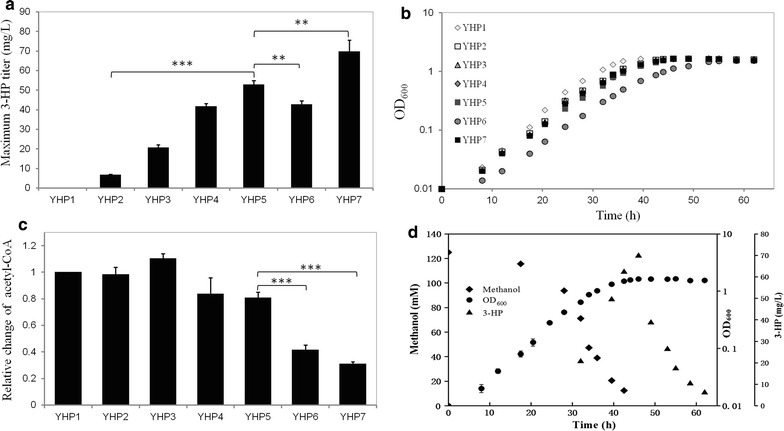
3-HP production in engineered *M. extorquens* AM1.** a** Maximum 3-HP titer of engineered strains;** b** effect of Mcr overexpression on cell growth;** c** comparison of acetyl-CoA pool in engineered strains at the exponential phase. The y axis is the ratio of intracellular acetyl-CoA between the strains of YHP2, YHP3, YHP4, YHP5, YHP6 and YHP7 to the strain YHP1 respectively. The average value for the YHP1 strain was set to 1;** d** methanol consumption, growth curve and 3-HP degradation at the transition from exponential phase to stationary phase in the YHP7 strain. YHP1 (*M. extorquens* AM1/pJY80), YHP2 [*M. extorquens* AM1/pJY80 (P_*meta1_3616*_::*mcr*)], YHP3 [*M. extorquens* AM1/pJY80 (P*coxB*::*mcr*)], YHP4 [*M. extorquens* AM1/pJY80 (P*tuf*::*mcr*)], YHP5 [*M. extorquens* AM1/pJY80 (P_*mxaF*_::*mcr*)], YHP6 [*M. extorquens* AM1/pJY80 (P_*mxaF–mxaF*_::*mcr*)], YHP7 [BHBT5/pJY80 (P_*mxaF*_::*mcr*::P_meta1_3616_::mcr_550–1219_)]. The data presented for each strain were the mean calculated from three independent biological replicates (***P ≤ 0.001; **P ≤ 0.01; t test)

In order to produce 3-HP in *M. extorquens* AM1, we constructed a malonyl-CoA pathway by overexpressing the *mcr* gene from *Chloroflexus aurantiacus* DSM 635 encoding a bifunctional enzyme with alcohol dehydrogenase and aldehyde dehydrogenase activities under the control of a weak promoter (P_*meta1_3616*_), yielding the YHP2 strain. As shown in Fig. 2a, YHP2 generated 6.8 mg/l of 3-HP when grown on methanol in shake flasks. To further improve the production level, the expression of *mcr* was tuned with three stronger promoters [32, 39]. The titer of 3-HP was increased in a promoter strength-dependent manner. The strain overexpressing *mcr* with the strongest promoter *mxaF* (YHP5) produced 52.8 mg/l of 3-HP, which was 7.8-fold higher than that of strain YHP2. Moreover, the specific growth rate and biomass yield were not significantly different among the strains of YHP3, YHP4 and YHP5 (Fig. 2b and Table 1), suggesting that gradual increase of Mcr expression did not lead to significant metabolic burden. To further improve expression of *mcr*, we constructed a dual-promoter of *mxaF*–*mxaF*. The strength of this promoter was 1.2-fold higher than that of P_*mxaF*_ as shown in Additional file 1: Table S1. Surprisingly, the titer in the YHP6 strain was decreased by 1.2-fold compared to the YHP5 strain, and specific growth rate was also decreased by 16.7% (Fig. 2b and Table 1). It has been reported that N-terminal region of Mcr (Mcr-N, amino acids 1–549) and the C-terminal region of Mcr (Mcr-C, amino acids 550–1219) were functionally distinct (shown in Fig. 1) and Mcr-C had the lower enzymatic activity. [11]. We then inserted the *mcr*_550–1219_ sequence promoted by P_*meta1_3616*_ after the full *mcr* on the plasmid and transferred it into the BHBT5 strain, an adaptive evolved strain which has been isolated from *M. extorquens* AM1 with faster growth rate [33]. The new recombinant strain YHP7 produced 69.8 mg/l of 3-HP, representing 1.3-fold improvement over that of the YHP5 strain, while the specific growth rate was not changed compared to the YHP5 strain (Fig. 2b and Table 1). Acetyl-CoA, a precursor of the malonyl-CoA pathway was further analyzed and found to be 1.9- and 2.9-fold lower in the YHP6 and YHP7 strain compared to the YHP5 strain (Fig. 2c). Notably, when the cells reached the stationary growth phase, 3-HP was rapidly reduced to lower than 10.0 mg/l within 10 h in either the YHP5 strain (Additional file 2: Figure S1) or the YHP7 strain (Fig. 2d). Therefore, it is important to investigate the potential mechanism of 3-HP degradation in *M. extorquens* AM1.

**Table 1:** Specific growth rate in engineered *M. extorquens* AM1

Strains	Specific growth rate (h^−1^)^a^
YHP1	0.114 ± 0.004
YHP2	0.104 ± 0.005
YHP3	0.101 ± 0.003
YHP4	0.098 ± 0.003
YHP5	0.096 ± 0.002
YHP6	0.080 ± 0.004
YHP7	0.098 ± 0.003

^a^Values represent the averages with standard deviations obtained from three biological replicates

### Discovery of intermediates involved in 3-HP assimilation

**Fig. 3:**
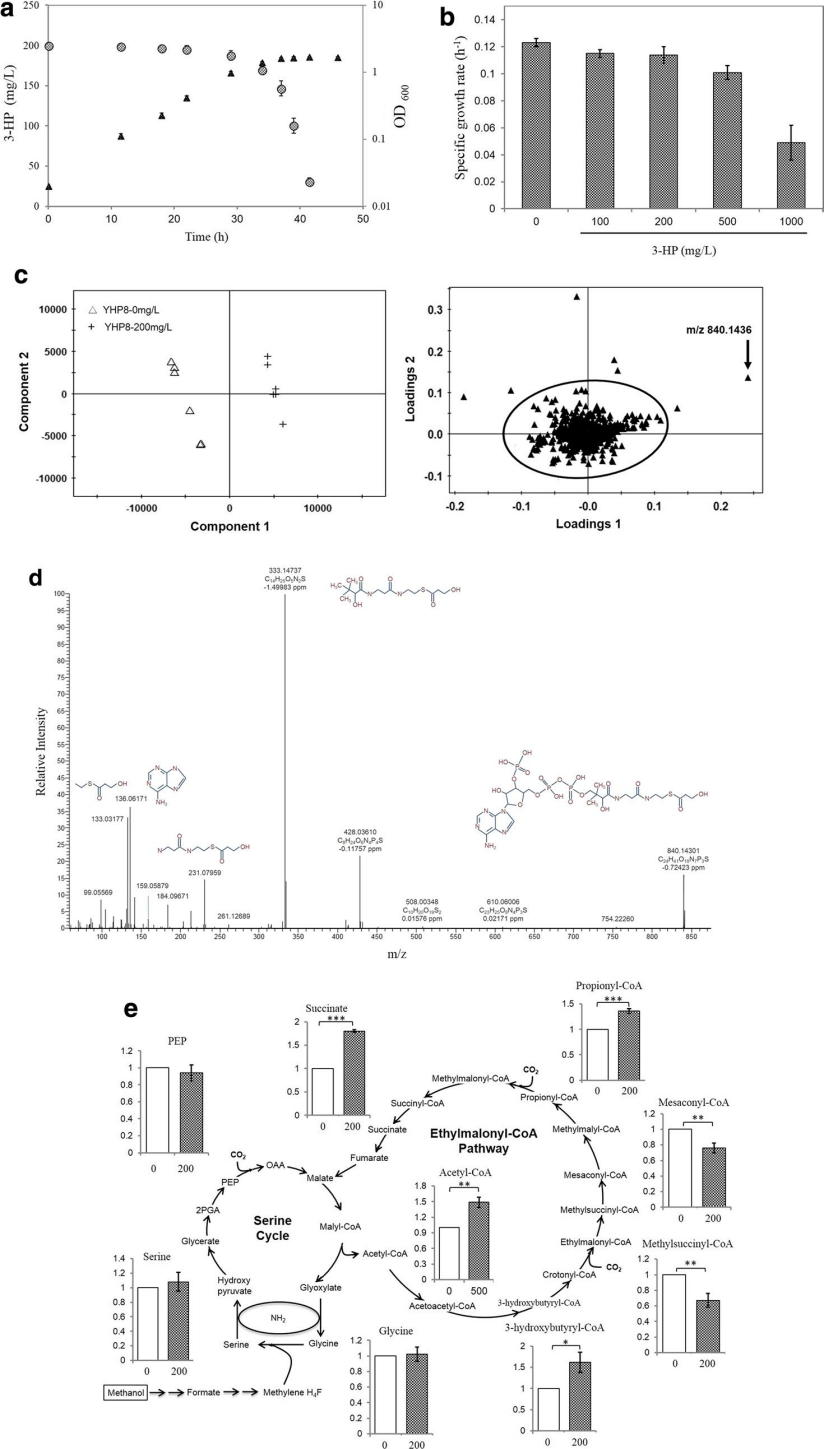
Discovers the metabolite change related to 3-HP assimilation.** a** 3-HP degradation (circle) and growth profile (triangle) in the YHP8 strain with the addition of 200 mg/l 3-HP. YHP8 (BHBT5/pJY80);** b** comparison of the specific growth rate in the YHP8 strain with the addition of different amounts of 3-HP;** c** untargeted metabolomics analysis between strain YHP8 with and without the addition of 3-HP. The metabolomic data was processed by PLS-DA. Data was acquired from six independent biological replicates;** d** the fragmental structures of m/z 840.1436 was predicted by MassFrontier. The mass accuracy between the theoretical mass and experimental mass is shown below the structure;** e** comparison of intracellular metabolites involved in the serine cycle and EMC pathway between the YHP8 strain with and without the addition of 3-HP. The y axis is the ratio of the intermediates between strain YHP8 with and without the addition of 3-HP. X axis is the concentration (mg/l) of 3-HP in the medium. The average value for the control strain was set to 1. Data show the mean with error bars indicating standard deviation calculated from three independent biological replicates (***P ≤ 0.001; **P ≤ 0.01; *P ≤ 0.05, t test)

Different concentrations of 3-HP were added to the culture of the YHP8 strain carrying empty plasmid pJY80. As shown in Fig. 3a, the extracellular 3-HP concentration decreased rapidly from 200.0 mg/l to less than 40.0 mg/l during the transition from exponential to stationary growth phase, and the growth rates was not significantly changed with the addition of 3-HP up to 200.0 mg/l (Fig. 3b). To clarify the fast degradation of 3-HP, we compared the metabolic profiles between the YHP8 with and without the addition of 3-HP by LC–MS and GC–MS. The samples were harvested in the late of exponential phase. For untargeted metabolome analysis, a partial least squares discrimination analysis (PLS-DA) method was used to analyze data. Examination of the scores plot in Fig. 3c showed the YHP8 strain with the addition of 200.0 mg/l of 3-HP was clearly separated from the YHP8 strain without the addition of 3-HP. The loading plot further revealed that the main features responsible for this separation were the scattered dots outside the circle. Among seven features, one feature with *m/z* 840.1436 was solely synthesized in the YHP8 strain with the addition of 200.0 mg/l 3-HP. Its detailed MS/MS fragmental information is shown in Fig. 3d and it is identified as 3-hydroxypropionyl-CoA (3-HP-CoA). Targeted metabolic profiling suggested that the amounts of several EMC intermediates including propionyl-CoA, succinate, acetyl-CoA and 3-hydroxybutryryl-CoA were increased 1.4-, 1.8-, 1.5- and 1.6-fold in the YHP8 strain in the presence of 200.0 mg/l of 3-HP compared to the control (Fig. 3e). Other metabolites such as glycine and serine in the serine cycle maintained a similar level with or without 3-HP. In addition, mesaconyl-CoA and methylsuccinyl-CoA were found to be slightly decreased by 0.8- and 0.7-fold. Overall, these results suggested that 3-HP might be converted to 3-hydroxypropionyl-CoA, which was reassimilated by the EMC pathway.

## ^13^C-labeling experiment demonstrates a reductive route as the major pathway for 3-HP assimilation

**Fig. 4:**
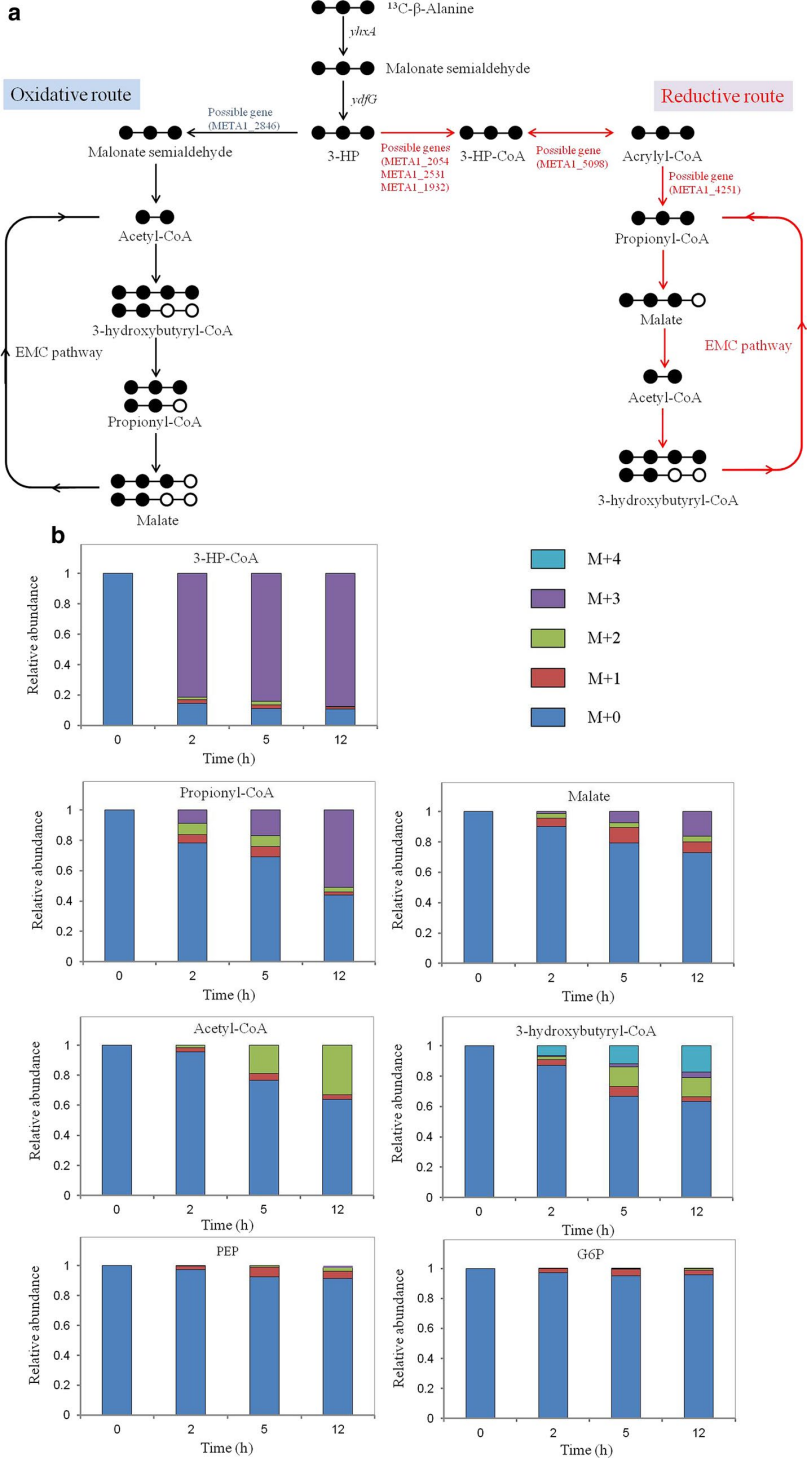
^13^C-labeling experiment demonstrates that the reductive route was mainly responsible for 3-HP degradation. Genes yhxA and ydfG encoding β-alanine-pyruvate aminotransferase and 3-hydroxypropanoate dehydrogenase respectively.** a** Predicted major labeling patterns from ^13^C-labeled β-alanine through the reductive route and oxidative route;** b** time course of ratios of isotopomers of some intermediates after the switch over from ^12^C-β-alanine to ^13^C-β-alanine in engineered strain YHP9 grown on ^12^C-methanol. M + 0, unlabeled; M + 1, singly labeled; M + 2, doubly labeled; M + 3, triply labeled; M + 4, quadruply labeled. YHP9 [BHBT5/pJY80 (P_mxaF_::yhxA-ydfG)]. Data was calculated from three independent biological replicates. Possible genes involved in reassimilation through the reductive route were added accordingly

In order to verify this hypothesis, the dynamic ^13^C-incorporation of intermediates was studied through a tracing experiment. ^13^C-labeled 3-HP is not commercially available but labeled β-alanine is, so we first constructed a β-alanine pathway by overexpressing genes *yhxA* and *ydfG* in the BHBT5 strain to generate 3-HP [10]. As shown in Additional file 2: Figure S2, 32.1 mg/l of 3-HP was produced in the YHP9 strain with the addition of 1 g/l β-alanine. Then a ^13^C-tracing experiment was carried out by switching from ^12^C-β-alanine to ^13^C-β-alanine in the middle of exponential phase as shown in Additional file 2: Figure S3. Major possible labeling patterns are shown in Fig. 4a. Triply labeled 3-HP-CoA was generated with 81.5% of the total pool within 2 h, demonstrating that triply labeled 3-HP was converted to 3-HP-CoA (Fig. 4b). Triply labeled propionyl-CoA appeared later, followed by triply labeled malate, which were 51.0 and 16.2% of the total pool up to 12 h, respectively. Doubly labeled acetyl-CoA was detected from 5 h, and increased to a total pool of 33.0% at 12 h. In addition, quadruply labeled 3-hydroxybutyryl-CoA was generated from the combination of two doubly labeled acetyl-CoA. Phosphoenolpyruvate (PEP) and glucose 6-phosphate/fructose 6-phosphate (G6P/F6P) were not obviously labeled. These data provide metabolic proof of 3-HP reassimilation through the reductive route coupled with the EMC pathway. The relatively low ^13^C-incorporation into propionyl-CoA, malate, acetyl-CoA and 3-hydroxybutyryl-CoA can be explained by the additional synthesis of unlabeled intermediates via the EMC pathway.

### Enzymatic assay to specifically and quantitatively detect acrylyl-CoA

**Fig. 5:**
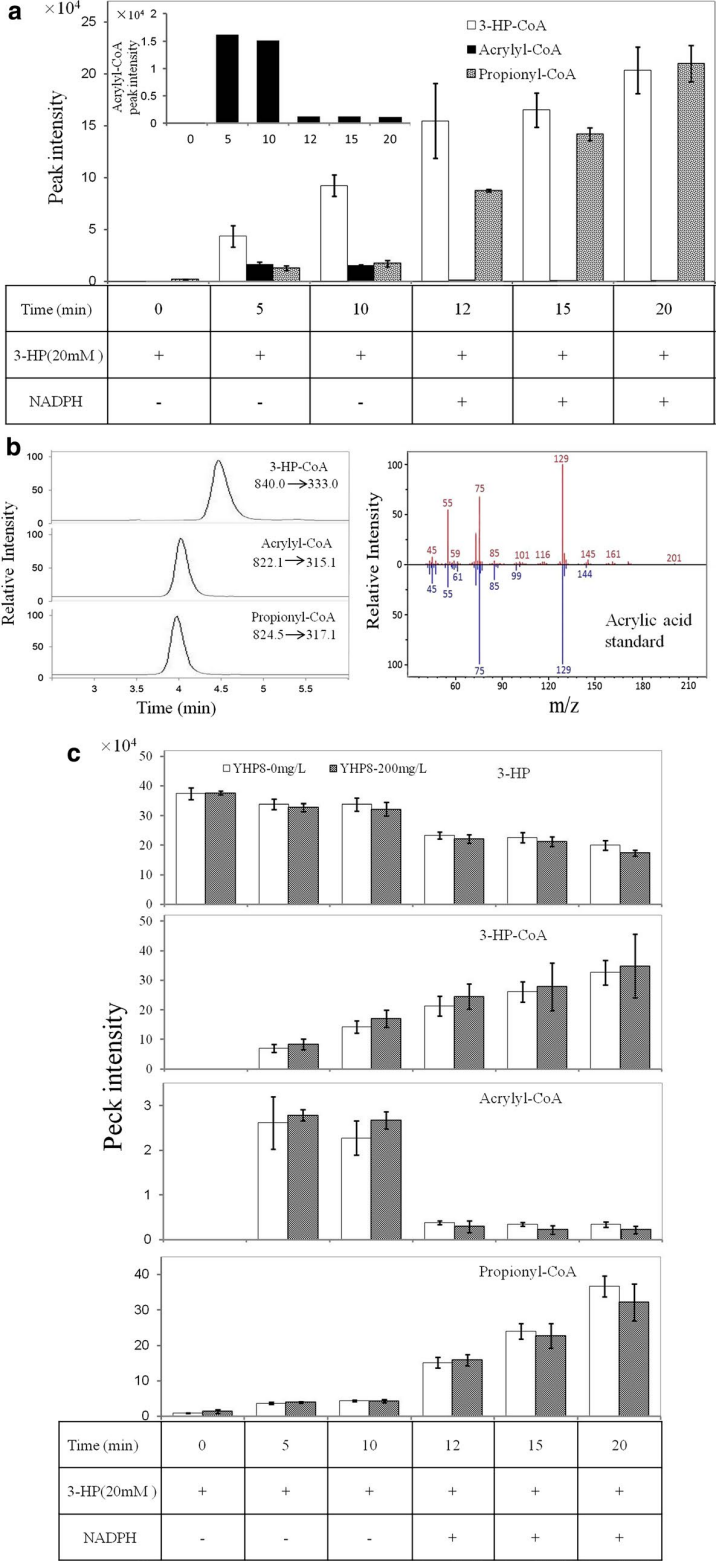
In vitro enzymatic assay detects the acrylyl-CoA production and effect of 3-HP on acyl-CoAs accumulation.** a** Analysis of acyl-CoAs formed during the reductive conversion of 3-HP by cell extracts of strain YHP8 grown on methanol. Shown are data for the reaction mixture for 10 min before the addition of NADPH and for 10 min after the addition of NADPH;** b** the extracted ion chromatograms of 3-HP-CoA, acrylyl-CoA and propionyl-CoA analyzed by LC–MS, and a comparison of mass fragments of acrylic acid on GC–MS between the experimental data (red) and NIST data (blue);** c** comparison of acyl-CoAs accumulation and 3-HP consumption by cell extracts of strain YHP8 with and without the addition of 20 mM 3-HP. YHP8 (BHBT5/pJY80). Data show the mean with error bars indicating standard deviation calculated from three independent biological replicates

Surprisingly, acrylyl-CoA was not detected in either the metabolomic analysis or ^13^C-labeling experiments but was known to be involved in natural assimilation of 3-HP in a previous report [14]. This nondetection is possibly due to the decomposition of acrylyl-CoA during the extraction process. An in vitro enzymatic assay was conducted to demonstrate if acrylyl-CoA was generated during 3-HP reassimilation in *M. extorquens* AM1. As shown in Fig. 5a, b and Additional file 2: Figure S4, 3-HP-CoA was significantly accumulated at 5 min after the addition of 20 mM 3-HP into the crude cell extracts from the YHP8 strain. In the meantime, a metabolite with MRM of *m/z* 822.1 to 315.1 was found to be accumulated at 5 and 10 min before the addition of NADPH, which had two *m/z* less than the parental ion of propionyl-CoA (Fig. 5b). The acid derivative of this metabolite was further analyzed and confirmed as acrylic acid by GC–MS (Fig. 5b). When NADPH was initially omitted from the assay mixture, the main product was 3-HP-CoA (Fig. 5a), which was consistent with the previous study that the ratio of 3-HP-CoA to acrylyl-CoA at equilibrium was greater than 50:1 [14]. Acrylyl-CoA was found to be quickly decreased after the addition of NADPH at 12 min and propionyl-CoA was increased accordingly, suggesting the acrylyl-CoA was rapidly reduced to propionyl-CoA. Moreover, the pre-addition of 200.0 mg/l 3-HP to the YHP8 strain did not change the consumption pattern of 3-HP and production patterns of 3-HP-CoA, acrylyl-CoA and propionyl-CoA, indicating that the activation of the reductive route was unlikely due to the presence of 3-HP (Fig. 5c). Together, these results clearly indicate that 3-HP was mainly reduced to propionyl-CoA through 3-HP-CoA and acrylyl-CoA as intermediates.

### Deletion of genes in 3-HP assimilation

**Fig. 6:**
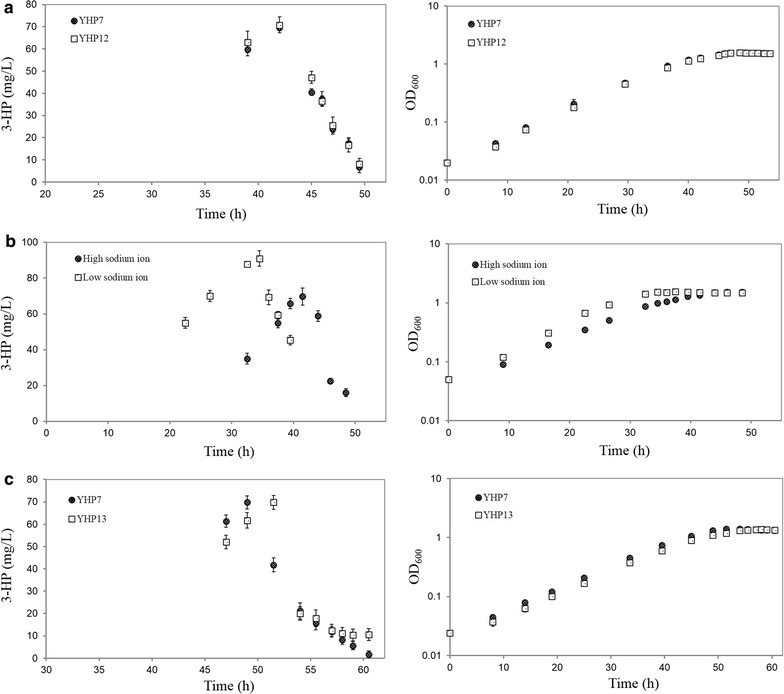
3-HP degradation and cell growth in the deletion mutants.** a** Comparison of the YHP7 strain and the YHP12 strain deleting the dctA gene;** b** effect of sodium ions on 3-HP degradation and cell growth of strain YHP7. High and low sodium ions are 972 and 16 μM in minimal medium respectively;** c** comparison of the YHP7 strain and the YHP13 strain deleting the META1_4251 gene. Data show the mean with error bars indicating standard deviation calculated from three independent biological replicates. YHP7 [BHBT5/pJY80 (P_mxaF_::mcr::P_meta1_3616_::mcr_550–1219_)], YHP12 [BHBT5 ΔMETA1_3271/pJY80 (P_mxaF_::mcr::P_meta1_3616_::mcr_550–1219_)], YHP13 [BHBT5 ΔMETA1_4251/pJY80 (P_mxaF_::mcr::P_meta1_3616_::mcr_550–1219_)]

To determine the possible genes involved in 3-HP reassimilation, we firstly knocked out *dctA* (META1_3271) encoding a putative protein transporter responsible for the uptake of C3 to C5 acids (e.g. pyruvic acid, succinic acid and methylsuccinic acid), then analyzed the 3-HP production in the deletion mutant YHP12 (YHP10 carrying pYM07). As shown in Fig. 6a, the deletion of *dctA* did not result in an obvious change of 3-HP degradation and cell growth. Since it might exist additional DctA system and DctA transporter has been reported to be sodium ion dependent [40], we hypothesized that reducing sodium concentration in the medium might effect the degradation. As shown in Fig. 6b, it indicated that a decrease of sodium ion in the culture medium did not prevent the degradation either, although the cell growth rate was increased by 1.2-fold and 3-HP production level was improved to 91.0 mg/l. Moreover, we blasted the previously reported enzymes involved in catalyzing two steps of 3-HP reduced to propionyl-CoA against the proteome of *M. extorqu*ens AM1. As summarized in Table 2, several homologues of acrylyl-CoA reductase and CoA-transferase/CoA-synthetase were identified in the genome of *M. extorquens* including META1_4251 and META1_2054. No growth change was observed for the deletion of gene META1_4251 (Fig. 6c). Moreover, the mutant strain YHP13 showed a similar decreasing rate of 3-HP degradation with the YHP7 strains in early stationary phase, but a slower decrease was observed when the 3-HP titer was reduced to 15.0 mg/l. In addition, it was not possible to obtain a deletion of the putative CoA-transferase gene META1_2054 growing on different carbon sources, suggesting that a null mutant is lethal for *M. extorquens* AM1. The protein encoded by META1_2054 was then expressed in *E. coli* BL21 (DE3) and purified. An in vitro assay showed much less 3-HP-CoA production than that in the crude enzymatic assay (Additional file 2: Figure S5).

**Table 2:** Proteins of *M. extorquens* AM1 showing highest homology by BLAST analysis

Description	Protein ID	Gene locus taq	Identity (%)	References
Putative acrylyl-CoA reductase^1,2,3^	WP_003599507.1	META1_4251	59^1^, 54^2^, 52^3^	[14, 50]
Enoyl-CoA hydratase^4^	WP_003602251.1	META1_5098	51	[51]
Putative acyl CoA: acetate/3-ketoacid CoA transferase^5,6,7^	WP_003598031.1	META1_2054	51^4^,44^5^, 44^6^	[51]
Acetyl-CoA synthetase^8,9^	WP_012752884.1	META1_2531	67^7^, 49^8^	[51]
Putative propionate-CoA ligase (propionyl-CoA synthetase)^10^	WP_012752709.1	META1_1932	62	[51]
NAD(P)-dependent oxidoreductase^11^	WP_012753094.1	META1_2846	42	[51]

Homology to ^1^ *Rhodobacter sphaeroides* (AcuI GenBank number ABA77575), ^2^ *Ruegeria pomeroyi* DSS-3 (SPO_1914 GenBank number AAV95191) and ^3^ *Escherichia coli* K-12 substrain MG1655 (YhdH GenBank number NP_417719); homology to ^4^ *Clostridium acetobutylicum* ATCC 824 (short-chain-enoyl-CoA hydratase GenBank number NP_349318); homology to ^5^ *Clostridium propionicum* (YdiF GenBank number Q9L3F7), ^6^ *Cupriavidus necator *(YdiF GenBank number Q0K874), and ^7^ *Escherichia coli *(YdiF GenBank number P37766); homology to ^8^ *Escherichia coli* (acetyl-CoA synthetase GenBank number P27550) and ^9^ *Salmonella typhimurium* (propionate-CoA ligase GenBank number P55912); homology to ^10^ *Ruegeria pomeroyi* (propionate-CoA ligase GenBank number Q5LPB4); homology to ^11^ *Pseudomonas denitrificans* ATCC 13867(3-hydroxyisobutyrate dehydrogenase GenBank number AGI23197)

## Discussion

*Methylobacterium extorquens* AM1 has been considered as a potential platform strain for industrial production of valuable chemicals such as mevalonate, 1-butanol and 2-hydroxyisobutyrate [30, 32, 33, 41]. In this work, we first optimized a 3-HP synthetic pathway in *M. extorquens* AM1, and then focused on the demonstration of the mechanism of 3-HP reassimilation.

It has been reported that tuning of gene expression levels was critical for proper functioning of a heterologous synthetic pathway in *M. extorquens* AM1. For instance, Hu et al. found that the strain expressing the *adhE2* and *ter* from a promoter of intermediate strength produced the highest 1-butanol [32]. In our case, four different promoter strengths were tested and the strongest promoter *mxaF* was shown to generate the highest 3-HP, comparable with the preliminary titer of other engineered microorganisms [5]. The pool size of precursor acetyl-CoA was similar between the YHP5 strains and the other three recombinant strains, implying that upstream metabolic fluxes were not significantly affected by the introduction of the malonyl-CoA pathway. Moreover, overexpressing *mcr*_550–1219_ resulted in further improvement of 3-HP production and decrease of the acetyl-CoA pool size, suggesting that this synthetic pathway drew more acetyl-CoA flux into 3-HP synthesis and the supply of acetyl-CoA may become insufficient. In the YHP6 strain carrying dual strongest promoters of *mxaF*–*mxaF*, the titer of 3-HP and the pool of acetyl-CoA were both lower than that in the YHP5 strain. This was presumably because high expression of Mcr could cause metabolic imbalance and in turn interrupt the entire flux distribution. In order to pull more flux to acetyl-CoA, we overexpressed the gene *pyk* encoding pyruvate kinase which was predicted to lead to higher relative fluxes into acetyl-CoA in *M. extorquens* AM1 [42]. However, the improvement of 3-HP production was not observed (Additional file 2: Figure S6). In addition, the constructed malonyl-CoA pathway led to the net consumption of 2 molecules of NADPH per 1 molecule of 3-HP produced. In *M. extorquens* AM1 grown with methanol, cell growth is limited by reducing power [36], suggesting that an insufficient supply of NADPH is another bottleneck for 3-HP production. Further multiple genes manipulation for improving acetyl-CoA and reducing power supply is necessary to enhance the 3-HP production in *M. extorquens* AM1. In addition, a partial β-alanine pathway has been constructed to be able to produce the 3-HP (Additional file 2: Figure S2), it would be interesting to combine full β-alanine pathway with malonyl-CoA pathway to figure out whether the combined pathways would improve the titer of 3-HP further.

Recent reports have demonstrated that 3-HP was steadily produced by utilizing either the malonyl-CoA pathway or the β-alanine pathway [5–10, 43]. However, in *M. extorquens* 3-HP was rapidly degraded upon the transition from exponential to stationary growth phase. The addition of 3-HP into the medium of the cell culture further demonstrated that 3-HP degradation was lower than the release during exponential phase but higher during stationary phase. This degradation phenomenon was also observed in the recombinant *P. denitrificans*, *Meyerozyma guilliermondii* and *Rhodococcus erythropolis* [15, 44, 45]. Lee et al. demonstrated that the genes *hpdh* and *hbdh* encoding 3-HP dehydrogenase and 3-hydroxyisobutyrate dehydrogenases in *P. denitrificans* were mainly responsible for 3-HP degradation through the oxidative route, in which 3-HP was first oxidized to malonate semialdehyde and then metabolized to acetyl-CoA [17]. However, neither untargeted nor targeted metabolome analysis detected the accumulation of malonate semialdehyde, which might be due to its instability in the extraction or low pool abundance in cell. But 3-HP-CoA, propionyl-CoA and its downstream intermediates were observed to build up in the cell, suggesting 3-HP was reduced to 3-HP-CoA via the reductive route and then reassimilated by the EMC pathway.

Previously we have demonstrated a highly efficient pyrophosphate-mediated glycolytic pathway for methane assimilation in *Methylomicrobium alcaliphilum* strain 20Z by tracing the pyruvate ^13^C-labeling pattern [46]. Here, if 3-HP reassimilation flux through the reductive pathway produced triply labeled 3-HP-CoA and propionyl-CoA, and the latter enters the EMC pathway, malate would maintain the same labeled pattern and have faster ^13^C-incorporation than its downstream metabolite, acetyl-CoA. If 3-HP was reassimilated by the oxidative route, it would be expected to generate more amounts of doubly labeled acetyl-CoA and fewer amounts of doubly and triply labeled propinyl-CoA in a time course. Our results demonstrated that both routes operated but the reductive route functioned as the major one for reassimilating the 3-HP based on two possible explanations. Firstly 3-HP-CoA and propionyl-CoA were more significantly labeled compared to acetyl-CoA, proposing that reductive route flux was higher than oxidative route. Secondly by 5 and 12 h, the labeled pool of acetyl-CoA was much higher than its precursor of malate, suggesting part of labeled acetyl-CoA might come from the oxidative route. Moreover, the enzymatic assay not only confirmed that the reductive route for 3-HP assimilation in *M. extorquens* AM1 was through acrylyl-CoA as intermediates, but also suggested that the genes encoding enzymes for the reductive route were unlikely able to be induced by 3-HP. The latter finding differed with the previous observation that the degradation of 3-HP by the oxidative route in *P. denitrificans* was increased greatly when cells were exposed to 3-HP [47]. In that study, the transcription of *hpdh* and *hbdh* involved in the oxidative route was found to be induced by a LysR-type transcriptional regulator binding with 3-HP molecule.

*Methylobacterium extorquens* AM1 harbors a predicted DctA dicarboxylic acid transporter, which has been proposed to uptake C4 or C5 dicarboxylic acid and even C3 pyruvate [35, 38, 48]. Therefore, a knock out mutant of the *dctA* homologue (META1_3271) is expected to have an impact on the production of 3-HP. Unfortunately, *dctA* mutant was still able to degrade the 3-HP, which may either be due to an incomplete disruption of acid transport or to the existence of at least one additional system that can transport 3-HP into the cell [48, 49]. In addition, the transport function of some DctA examples has been shown to be sodium ion dependent, and reduced sodium ion has been demonstrated to block the reuptake of mesaconic acid and methylsuccinic acid in engineered *M. extorquens* AM1 [48]. However, in our study 3-HP degradation was not decreased when the sodium ion was reduced to 60-fold lower, suggesting either DctA is not playing a role in 3-HP degradation or it is independent of sodium ion.

An acrylyl-CoA reductase (AcuI) catalyzing the reduction of acrylyl-CoA to propionyl-CoA has been identified in *Ruegeria pomeroyi* and *R. sphaeroides* (Table 2) [50]. The *acuI* mutant was unable to grow on 3-HP as sole carbon source in *R. sphaeroides* [14]. The deletion of gene META1_4251 encoding a protein with 59% amino acid sequence identity to AcuI of *R. sphaeroides* resulted in slower degradation of 3-HP in late stationary phase. One possible explanation is that low accumulation of acrylyl-CoA in the beginning of stationary phase would not be capable of disrupting the reaction equilibrium between 3-HP-CoA and acrylyl-CoA, but high accumulation of acrylyl-CoA would generate a strong feedback to shift the equilibrium against acrylyl-CoA production. Hence, we propose that META1_4251 is involved in the third reaction of 3-HP assimilation. In addition, acyl-CoA transferase or acetyl-CoA/propionyl-CoA synthetase was reported to be responsible for converting 3-HP to 3-HP-CoA in engineered *E. coli* for the production of acrylic acid (Table 2) [51]. An attempt was made to delete the gene META1_2054 encoding a putative acyl-CoA transferase with 51% amino acid sequence identity to YdiF of *Cupriavidus necator*. However, we failed to obtain the null mutant, suggesting this gene might have an essential function for *M. extorquens* AM1 growth. The purified protein was shown to exhibit poor catalysis with 3-HP as a substrate, suggesting that META1_2054 was unlikely a key gene for the first reaction in the reductive route. Therefore, once other genes are identified and biochemically characterized, it may become clearer how *M. extorquens* AM1 reassimilates 3-HP and will prevent the degradation and enhance product formation rate.

## Conclusions

The engineered *M. extorquens* AM1 demonstrated the production of 3-HP on methanol with a titer of 6.8 mg/l in shake flask cultivation, which was further improved over tenfold by increasing the promoter strength and copy number of *mcr*_550–1219_. Although further strain optimization is required to make this system industrially relevant, metabolic engineering precedents exist that have resulted in similar magnitudes of increase [7]. It has been shown in engineered *P. denitrificans* that 3-HP was degraded through an oxidative route, in which 3-HP was first oxidized to malonate semialdehyde and then metabolized to acetyl-CoA [17, 18]. However, our metabolomics, ^13^C-labeling analysis, in vitro enzymatic assays and knockout experiment demonstrated that 3-HP was mainly reduced to 3-HP-CoA and then sequentially converted to acrylyl-CoA and propionyl-CoA during the growth transition in engineered *M. extorquens* AM1. This novel work makes a good start for bioconversion of methanol into economically important product of 3-HP.

## Methods

### Culture medium and condition

*Escherichia coli* strain Top10 used as a host to construct all recombinant plasmids was cultivated at 37 °C in Luria–Bertani (LB) medium. *M. extorquens* AM1 was routinely cultured in a minimal medium as described previously [52]. Briefly, the strains were grown in tube at 30 °C to the middle exponential phase, sub-cultured (0.5 ml) into 50 ml of minimal medium in 250 ml flasks, and then grown at 30 °C on rotary shaker at 200 rpm. Substrates and antibiotics were supplied at the following concentrations: methanol (125 mM), succinate (15 mM), 20 μg/ml tetracycline (Tet) and 50 μg/ml ampicillin (Ap). During the cultivation process, measurements of the cell growth were conducted in biological triplicate. To assess the growth rates in the presence of 3-HP, 0.5 ml of each culture was distributed into 50 ml fresh medium in 250 ml flasks containing different concentrations of 3-HP. The growth rates presented represent the mean plus STDEV calculated from triplicate biological replicates.

All chemicals were purchased from Sigma-Aldrich (St. Louis, MO, USA) unless otherwise specified. Millpore water (Billerica, MA, USA) was used for the preparation of all the media, buffers, standards, and sample solutions.

### Plasmids and strains construction

For constructing the malonyl-CoA pathway, the protein sequence of malonyl-CoA reductase (Mcr) was retrieved from GenBank accession number of YP_001636209. Gene coding for this enzyme was cloned from *Chloroflexus aurantiacus* DSM 635 genomic DNA. The strains and plasmids were listed in Table 3 and the primers were provided in Additional file 1: Table S2. Standard restriction enzyme digestion and ligation techniques were used to construct plasmids. The *mcr* gene was PCR amplified with PrimeSTAR HS DNA Polymerase and assembled into the *Hin*dIII–*Bam*HI restriction sites of pJY80 plasmid with different promoter regions. The strength of promoters was tested based on the construction of promoter fusion as described before [53]. The promoter fragments of *meta1_3616* and *coxB* from genomic *M. extorquens* AM1 DNA and *mxaF* from pCM80 plasmid were amplified by PCR. And the dual-promoter *mxaF*–*mxaF* was obtained by an overlapping PCR method [54]. The amplified fragments were then cloned into *Bam*HI–*Hin*dIII restriction sites of the promoter probe vector pCM130 [55]. Catechol dioxygenase (XylE) was assayed as described previously to evaluate the promoter strength [56]. For plasmid construction of the YHP7 strain, two PCR fragments of the promoter *meta1_3616* and *mcr*_550–1219_ were assembled into the plasmid of the YHP5 strain after full *mcr* fragment under a *mxaF* promoter. For constructing the β-alanine pathway, the protein sequence of β-alanine-pyruvate aminotransferase (YhxA) and 3-hydroxypropanoate dehydrogenases (YdfG) was retrieved from GenBank with the following accession number of EEL86940 and 12932746. Gene *yhxA* coding for the YhxA enzyme was synthesized into the vector pUC57 (GenScript, Nanjing, China) with codon usage optimized for expression in *M. extorquens* AM1. And gene *ydfG* coding for the YdfG enzyme was cloned from *E. coli* str.K-12 genomic DNA. As described above, the fragments of *yhxA* and *ydfG* were assembled into the *Hin*dIII–*Bam*HI restriction sites of pJY80 plasmid with the *mxaF* promoter to obtain pYM09 plasmid. All the plasmids were transformed into *M. extorquens* AM1 by electroporation as described before [57].

**Table 3:** Strains and plasmids used in this study

Strain or plasmid	Description	Source or references
Strains		
*M. extorquens* AM1	Wild-type, pink color, rifamycin-resistant strain	[21]
BHBT5	Adaptive evolved strain of *M. extorquens* AM1 with butanol tolerance, white color	[33]
YHP1	*M. extorquens* AM1/pJY80	This study
YHP2	*M. extorquens* AM1/pYM02	This study
YHP3	*M. extorquens* AM1/pYM03	This study
YHP4	*M. extorquens* AM1/pYM04	This study
YHP5	*M. extorquens* AM1/pYM05	This study
YHP6	*M. extorquens* AM1/pYM06	This study
YHP7	BHBT5/pYM07	This study
YHP8	BHBT5/pJY80	This study
YHP9	BHBT5/pYM09	This study
YHP10	BHBT5 ΔMETA1_3271	This study
YHP11	BHBT5 ΔMETA1_4251	This study
YHP12	YHP10/pYM07	This study
YHP13	YHP11/pYM07	This study
Plasmids		
pCM130	Low-background *xylE* promoter-probe vector, Tet^R^	[55]
pCM433	*sacB*-based allelic exchange vector; Ap^R^, Cm^R^, Tet^R^	[58]
pJY80	pCM80-based, no promoter P_*mxaF*_; Tet^R^	Lab storage
pYM02	pJY80 (P_*meta1_3616*_::*mcr*)	This study
pYM03	pJY80 (P_*coxB*_::*mcr*)	This study
pYM04	pJY80 (P_*tuf*_::*mcr*)	This study
pYM05	pJY80 (P_*mxaF*_::*mcr*)	This study
pYM06	pJY80 (P_*mxaF–mxaF*_::*mcr*)	This study
pYM07	pJY80 (P_*mxaF*_::*mcr*::P_*meta1_3616*_::mcr_*550–1219*_)	This study
pYM09	pJY80 (P_*mxaF*_::*yhxA-ydfG*)	This study
pYM10	pCM433 with upstream and downstream fragments of META1_3271	This study
pYM11	pCM433 with upstream and downstream fragments of META1_4251	This study

Ap^R^ ampicillin resistance, Cm^R^ chloramphenicol resistance, Tet^R^ tetracycline resistance

Allelic exchange was performed using the *sacB*-based vector pCM433 [58]. Briefly, the PCR product obtained corresponding to merged 600-bp upstream and downstream flanking regions of META1_3271 or META1_4251 from the adaptive strain of *M. extorquens* AM1 (strain BHBT5) [33] was inserted into the BglII and NdeI restriction sites of pCM433. The plasmids were then electroporated into the BHBT5 strain. Single-crossover mutants were selected using Tet resistance, and double-crossover mutants selected by growth on plates containing 5% w/v sucrose. Successful allele swapping was confirmed by diagnostic PCR with gene sequencing.

The restriction enzymes were purchased from Fisher Scientific (Pittsburgh, PA, USA). PrimeSTAR HS DNA Polymerase and T4 ligase were purchased from Takala (Dalian, China).

### 3-HP quantification

The culture medium containing 3-HP was collected by centrifuging at 10,000×*g* for 10 min and the supernatant was used for product analysis. The concentration of 3-HP was determined by LC–MS carried out on an Agilent LC-QQQ-MS system (Agilent 1290 Infinity-6460, Agilent Corp, SantaClara, CA, USA) in the negative-ion mode. 3-HP standard was purchased from Tokyo Chemical Industry (Tokyo, Japan). The sample was separated on an Agilent SB C18 column (100 × 2.1 mm, 1.8 μm). Mobile phase A consisted of 10% (v/v) buffer in water, while mobile phase B was 10% (v/v) buffer in acetonitrile. The buffer consisted of 200 mM formic acid adjusted to pH 4.0 with ammonium hydroxide solution. The linear gradient was as following: 0–3 min, 5–10% B; 3–5 min, 100% B; 5–7 min, 5% B. The flow rate was 0.2 ml/min and the column was set at 35 °C. The injection volume was 5 μl. The 3-HP titer was measured in biological triplicate. The significance of differences between the different engineered strains was determined by t tests (MS excel) with a p value less than 0.05 considered to be statistically significant.

### Measurement of methanol consumption

Methanol consumption was analyzed on gas chromatograph with a flame ionization detector (GC-FID7900, Tian Mei, Shanghai, China). The sample was separated on a RtxR-1 column (30 m × 0.25 mm, 0.25 μm, Restek, Bellefonte, USA). The inlet and FID temperatures were set at 200 and 220 °C. The temperature program was as follows: 60 °C with a hold time of 4 min, followed by an increase to 220 °C at a rate of 15 °C/min. Temperature was held at 220 °C for 5 min. 0.5 μl of sample was injected in split-less mode.

### Extraction and measurement of metabolites

Samples (20 ml) of the later exponential phase at the OD_600_ of 1.3 ± 0.1 were rapidly harvested by vacuum filtration using MILLEX-GP PES membrane filters (0.22 μm, 33 mm) and quickly washed with the growth medium as described before [59]. Extraction of metabolites was carried out as previously published for *M. extorquens* AM1 [60, 61]. Briefly, 10 ml of boiling water was added to a given sample and incubated at 100 °C for 10 min. The extracted cell suspension was cooled on ice for 5 min, then cell debris was removed by centrifugation at 5000×*g* for 5 min. The cell-free metabolite extract was centrifuged at 14,000×*g* for 8 min. The supernatant was dried in a rotational vacuum concentrator (Christ, Osterode, Germany) and stored at − 80 °C. For LC–MS analysis, each dried sample was dissolved in 100 μl of purified water. For GC–MS analysis, each sample was further derivatized in two steps. First, keto group were methoximated by adding 50 μl of methoxyamine solution [25 mg/ml methoxyamine hydrochloride in pyridine] and incubated at 60 °C for 30 min. Second, trimethylsilylation was performed by adding 50 μl of a TMS reagent (BSTFA/TMCS, 99:1) and incubated at 30 °C for 90 min.

Sugar phosphates and acyl-CoAs were measured by LC–MS analysis. The sample analyzed on either an Agilent LC-QQQ-MS system (Agilent 1290 Infinity-6460, Agilent Technologies, Santa Clara, CA, USA), an Agilent LC-QTOF (Agilent 1290 Infinity-6530B, Agilent Technologies, Santa Clara, CA, USA) or a LC-QExactive-MS system (Thermo Fisher Scientific, Waltham, Massachusetts, USA). For LC-QQQ-MS, multiple reaction monitoring (MRM) precursor/product ion pairs were carried out as before [60]. For LC-QTOF, the *m/z* range was set to 50–1200 in centroid mode with a scan rate of 1.5 spectra/s. The ESI conditions were as follows: capillary voltage of 4000 V, fragmentor of 135 V, gas temperature of 300 °C, nebulizer of 35 psig, gas flow of 10 l/min. For LC-QExactive-MS system, spray voltage was set to + 3.2 kV, sheath gas pressure was 35 arb, aux gas pressure was 10 arb, capillary temperature was 320 °C and heater temperature was 350 °C. Full MS (Resolution 70,000) and MS2 (Resolution 17,500) were carried out with the scan range from *m/z* 100 to 1500. The sample was separated on an AdvanceBio Glycan column (150 × 2.1 mm, 1.8 μm; Agilent, Santa Clara, CA, USA). Mobile phase A consisted of 100 mM ammonium formate in water adjusted to pH 4.5 with ammonium hydroxide solution, while mobile phase B was 100% acetonitrile. The linear gradient was as following: 0–2 min, 75–71% B; 2.1–6 min, 65% B; 6–7 min, 65–75% B; 7–12 min, 75% B. The flow rate was 0.2 ml/min and the injection volume was 5 μl.

Amino acids and carboxylic acids were determined by GC–MS. The derivatized samples were analyzed on Agilent 5975B/6890N GC–MS instrument (Agilent Technologies, Santa Clara, CA, USA). The column was a HP-5MS (30 m × 0.25 mm × 0.25 μm film; Restek, Bellefonte, PA, USA). Ultra high purity helium was used as the carrier gas in a constant flow mode of 1 ml/min, and 1 μl of a given sample was injected in split-less mode via an Agilent 7890 autosampler. The inlet and transfer line temperatures were set at 280 °C. The temperature program started at 60 °C with a hold time of 0.25 min, and then increased at 5 °C/min to 280 °C with a hold time of 10 min at 280 °C. The ion source and quadrupole temperatures were set to 250 and 150 °C, respectively. Mass spectra were collected from *m/z* 40 to 500 at a rate of 3 spectra/s after a 4.5 min solvent delay.

### Metabolomics data processing

For the targeted metabolome, the data were presented as the mean of three independent biological replicates. For the untargeted metabolome, six independent biological samples were collected. LC–MS data was converted into mzML format using MS Convert software [62]. Data preprocessing and statistical analysis were performed with MZmine 2.11 and SIMCA-P 11.5 (Umetrics, Umea, Sweden) [63, 64]. The structure of metabolites was identified with MassFrontier 7.0 [65].

### Dynamic ^13^C-labeling analysis

^13^C-labeling analysis was performed by the protocol reported before with a slight modification [59]. 15 ml cells of the later exponential phase at the OD_600_ of 1.3 ± 0.1 were pre-cultured in minimal medium with ^12^C-methanol and 5 g/l ^12^C-labeled β-alanine, and then were rapidly passed through the membrane filter (0.22 μm, 47 mm, Sartorius). The filter was immediately removed and placed on an agar plate with ^12^C-methanol and ^12^C-labeled β-alanine for 30 min at 30 °C, then transferred to another agar plate with the same concentration of ^12^C methanol and ^13^C-labeled β-alanine for different times (i.e. 2, 5 and 12 h). Then the filter was immediately transferred to a 50 ml tube with liquid nitrogen for quenching. The sample was stored at − 80 °C freezer until it was ready for subsequent extractions. The metabolites were extracted and analyzed as the described above.

### Heterologous expression of META1_2054 in *E. coli* and protein purification

The META1_2054 gene was first amplified by PCR and cloned into the EcoRI and HindIII sites of pET.32M.3C. *E. coli* BL21 (DE3) harboring pET.32M.3C:: META1_2054 were grown in 100 ml of LB medium to an OD_600_ of 0.8 at 37 °C and were then induced with 200 μM isopropyl-β-d-thiogalactopyranoside (IPTG) for 18 h at 18 °C. Harvested cells were resuspended in 100 ml wash buffer A (50 mM NaH_2_PO_4_, 300 mM NaCl, pH 8.0). Crude cell extracts were obtained by passing the cells through One Shot cell disruptor (Constant Systems Ltd, United Kingdom) at 3.5 × 10^7^ psi, followed by 30 min of centrifugation at 14,000×*g* at 4 °C. The soluble fraction was then used for his-tagged purification by Ni-nitrilotriacetic acid (NTA) resin according to the manufacturer’s instruction (Pointbio, Shanghai, China). Then non-specifically bound proteins were removed from the column with wash buffer B (50 mM NaH_2_PO_4_, 300 mM NaCl, 40 mM imidazole, pH 8.0), while bound his-tagged protein were eluted with elution buffer (50 mM NaH_2_PO_4_, 300 mM NaCl, 100 mM imidazole, pH 8.0). Purified protein was verified by 12% SDS-PAGE, and the protein concentrations were determined according to the method of Bradford [66], using bovine serum albumin as a standard. To replace the elution buffer with enzyme reaction buffer (100 mM Tris–HCl, 5 mM MgCl_2_, 10 mM KCl, pH 8.0), the eluted solution was centrifuged through a centrifugal filter with a molecular cutoff of 30 kDa (Millipore, Billerica, MA), and the concentration of the enzyme was finally adjusted to 5 mg/ml by the reaction buffer.

### In vitro enzyme assays

*Methylobacterium extorquens* AM1 cell extracts was carried out as previously published with slight modification [32]. Briefly, 50 ml cells of the later exponential phase were harvested and then resuspended in 7 ml of 100 mM Tris–HCl (pH 8.0) buffer containing 5 mM MgCl_2_ and 10 mM KCl. Crude cell extracts were obtained by passing the cells through One Shot cell disruptor at 3.8 × 10^7^ psi. Dithiothreitol (2 mM) was added to cell extracts immediately after cell lysis. The reaction mixture (1 ml) was used to monitor the product formation of the reductive conversion of 3-HP [14]. The reaction mixture contained 100 mM Tris–HCl buffer at pH 8.0, 5 mM MgCl_2_, 10 mM KCl, 0.5 mM CoA, 3 mM ATP, 0.3 mM NADPH, 2 mM dithiothreitol and 0.3 mg cell extracts. The enzymatic reaction was started by the addition of 20 mM 3-HP but not NADPH to the mixture and stopped after 5 and 10 min respectively by transferring 100 μl of the reaction mixture into 4 μl 25% HCl. After 10 min, NADPH was added to the mixture, and the reaction was stopped at different time points as described above. The samples were centrifuged at 10,000×*g* for 5 min to remove the precipitated protein. Acyl-CoAs were analyzed by LC–MS as the described above. For assaying the META1_2054 coding enzyme, the product formation was measured as the described above except that NADPH was not add to the mixture.

## Additional files

**Additional file 1: Table S1.** XylE activities of promoter-*xylE* transcriptional fusions in wild-type *M. extorquens* AM1 grown on methanol. Table S2. Primers used in this study.

**Additional file 2: Figure S1.** 3-HP degradation at the transition from exponential phase to stationary phase of YHP5 [*M. extorquens* AM1/pJY80 (P_*mxaF*_ *::mcr*)]. Data was calculated from three independent biological replicates.** Figure S2.** 3-HP production in the YHP9 strain grown on methanol with the addition of β-alanine to the medium. X axis is the concentration (g/l) of β-alanine in the medium. YHP9 [BHBT5/pJY80 (P_*mxaF*_:: *yhxA*-*ydfG*)].** Figure S3.** ^13^C-tracing experiment was carried out by switching from ^12^C-β-alanine to ^13^C-β-alanine in the YHP9 strain.** Figure S4.** Control assay (i.e. no. 3-HP addition) did not detect the accumulation of 3-HP-CoA and acrylyl-CoA by cell extracts of strain YHP8 in a time course. Shown are data for the reaction mixture for 10 min before the addition of NADPH and for 10 min after the addition of NADPH. YHP8 (BHBT5/pJY80).** Figure S5.** Analysis of 3-HP-CoA formed during the reductive conversion of 3-HP catalyzed by the purified protein (META1_2054). The protein was expressed on pET.32M.3C in the strain *E. coli* BL21 (DE3).** Figure S6.** Growth curve and 3-HP production in the strain YHP14. The gene *pyk* was amplified from *M. extorquens* AM1 genome, the amplified fragments were then cloned into pYM05 plasmid to construct pYM12 (P_*mxaF*_ *::mcr*-*pyk*). Plasmid pYM12 was then transformed into *M. extorquens* AM1 by electroporation to obtain the strain YHP14 (*M. extorquens* AM1/P_*mxaF*_ *::mcr*-*pyk*).

## Abbreviations

3-HP: 3-hydroxypropionic acid
Mcr: malonyl-CoA reductase
Mcr-N: N-terminal region of Mcr
Mcr-C: C-terminal region of Mcr
EMC pathway: ethylmalonyl-CoA pathway
TCA cycle: tricarboxylic acid cycle
3-HP-CoA: 3-hydroxypropionyl-CoA
PEP: phosphoenolpyruvate
OAA: oxaloacetic acid
2PGA: 2-phosphoglyceric acid
G6P/F6P: glucose 6-phosphate/fructose 6-phosphate
XylE: catechol dioxygenase
YdfG: 3-hydroxypropanoate dehydrogenase
YhxA: β-alanine-pyruvate aminotransferase
AcuI: acrylyl-CoA reductase
IPTG: isopropyl-β-d-thiogalactopyranoside
PLS-DA: a partial least squares discrimination analysis
