# Supplementary material for: Production of 3-hydroxypropionic acid in engineered Methylobacterium extorquens AM1 and its reassimilation through a reductive route
Source: Microb Cell Fact. 2017 Oct 30;16:179. doi: 10.1186/s12934-017-0798-2 (PMC5663086; doi:10.1186/s12934-017-0798-2)
Supplement: Supplementary file 1 — Additional file 1: Table S1. XylE activities of promoter-xylE transcriptional fusions in wild-type M. extorquens AM1 grown on methanol. Table S2. Primers used in this study. [file 12934_2017_798_MOESM1_ESM.docx]

**Additional file 1**

Table S1 XylE activities of promoter-*xylE* transcriptional fusions in wild-type *M. extorquens* AM1 grown on methanol

| **Fusion** | **Mean activity of XylE (mU) ± SD^*^** |
| --- | --- |
| P*_meta1_3616_* :: *xylE* | 22.3±0.1 |
| P*_coxB_* :: *xylE* | 87.9±7.3 |
| P*_mxaF_* :: *xylE* | 347.6±7.4 |
| P*_mxaF-mxaF_* :: *xylE* | 407.9±4.0 |

^*^ One mU of XylE activity corresponds to 1 nmol produced catechol 2-hydroxymuconic semialdehyde min^-1^ ∙ mg^-1^ of protein. It was carried out in triplicate.

Table S2 Primers used in this study

| **Primers** | **Sequence (5’–3’)** |
| --- | --- |
| mcr_HindIII_fw | GAGAAGCTTCTCGAGATGAGCGGAACAGGACGACTG |
| mcr_BamHI_rev | GAGGGATCCCTCGAGTTACACGGTAATCGCCCGTC |
| P3616_NheI_fw | GAGGCTAGCCTCAAGGCACCGGCATC |
| P3616_HindIII_rev | GAGAAGCTTGCCGGCCACCCCACCGC |
| P3616_BamHI_fw | GAGGGATCCCTCAAGGCACCGGCATC |
| PcoxB_NheI_fw | GAGGCTAGCCGTATCCCCAGAGGCAGCC |
| PcoxB_HindIII_rev | GAGAAGCTTGTCCCCGCTTGGCTCCCCTG |
| PcoxB_BamHI_fw | GAGGGATCCCGTATCCCCAGAGGCAGCC |
| Ptuf_NheI_fw | GAGGCTAGCTGCTCGATCTCGGCGAGCG |
| Ptuf_HindIII_rev | GAGAAGCTTCGTGCAATCCTACCGCGGGAC |
| PmxaF_NheI_fw | GAGGCTAGCCCCGCTTGGTCGGGCCGCTTC |
| PmxaF_HindIII_rev | GAGAAGCTTGGCGTAATCATGGTCATAGCTG |
| PmxaF_BamHI_fw | GAGGGATCCCCCGCTTGGTCGGGCCGCTTC |
| PmxaF-PmxaF_OL_rev | GAAGCGGCCCGACCAAGCGGGTCTAGAGGCGTAATCATGGTCAT |
| PmxaF-PmxaF_OL_fw | ATGACCATGATTACGCCTCTAGACCCGCTTGGTCGGGCCGCTTC |
| P3616-mcrC_BamHI_fw | GAGGGATCCCTCAAGGCACCGGCATCGCTTC |
| P3616-mcrC_OL_rev | GCGCCGGTGGTGGCGCTGTGATGGTGATGATGGTGATGGAGATCTGCCATTCTAGAGCCGGCCACCCCACCGCTC |
| P3616-mcrC_OL_fw | GAGCGGTGGGGTGGCCGGCTCTAGAATGGCAGATCTCCATCACCATCATCACCATCACAGCGCCACCACCGGCGC |
| P3616-mcrC_EcoRI_rev | GAGGAATTCGAATTTACACGGTAATCGCCCGTCCGC |
| yhxA-ydfG_HindIII_fw | GAGAAGCTTATGGAGCTCATGATCGTCCAG |
| yhxA-ydfG_OL_rev | CTCCAGTTACTAAAACGATCATGGTATATCTCCTTTCAGAGCTGGGCCAGGCAC |
| yhxA-ydfG_OL_fw | GTGCCTGGCCCAGCTCTGAAAGGAGATATACCATGATCGTTTTAGTAACTGGAG |
| yhxA-ydfG_BamHI_rev | GAGGGATCCTTACTGACGGTGGACATTC |
| 3271_up_BglII_fw | GAGAGATCTCATTCACACCCTCGACGAGTTC |
| 3271_up_OL_rev | GGTGCGCCCTCCTGACCGGAGGGCGTTTCTCCCTGTCGGAC |
| 3271_down_OL_fw | GTCCGACAGGGAGAAACGCCCTCCGGTCAGGAGGGCGCACC |
| 3271_down_NdeI_rev | GAGCATATGACAGCTTGGTATCAACCGCC |
| 4251_up_BglII_fw | GAGAGATCTGTGCTACCGCCGCGTCGTGAC |
| 4251_up_OL_rev | TAAAATACGAAATGCCCCTGCGGTCCGCTCCTCCCGTCTCATG |
| 4251_down_OL_fw | CATGAGACGGGAGGAGCGGACCGCAGGGGCATTTCGTATTTTA |
| 4251_down_NdeI_rev | GAGCATATGATCATCCCTTTGGCGGGGTC |
| 2054_EcoRI_fw | GAGGAATTCATGAAGAAGAACAAGGTCATC |
| 2054_HindIII_rev | GAGAAGCTTTCAAGCGGCGGAAGCCACC |
