# Supplementary material for: Production of 3-hydroxypropionic acid in engineered Methylobacterium extorquens AM1 and its reassimilation through a reductive route
Source: Microb Cell Fact. 2017 Oct 30;16:179. doi: 10.1186/s12934-017-0798-2 (PMC5663086; doi:10.1186/s12934-017-0798-2)
Supplement: Supplementary file 2 — Additional file 2: Figure S1. 3-HP degradation at the transition from exponential phase to stationary phase of YHP5 [M. extorquens AM1/pJY80 (PmxaF ::mcr)]. Data was calculated from three independent biological replicates. Figure S2. 3-HP production in the YHP9 strain grown on methanol with the addition of β-alanine to the medium. X axis is the concentration (g/l) of β-alanine in the medium. YHP9 [BHBT5/pJY80 (PmxaF:: yhxA-ydfG)]. Figure S3. 13C-tracing experiment was carried out by switching from 12C-β-alanine to 13C-β-alanine in the YHP9 strain. Figure S4. Control assay (i.e. no. 3-HP addition) did not detect the accumulation of 3-HP-CoA and acrylyl-CoA by cell extracts of strain YHP8 in a time course. Shown are data for the reaction mixture for 10 min before the addition of NADPH and for 10 min after the addition of NADPH. YHP8 (BHBT5/pJY80). Figure S5. Analysis of 3-HP-CoA formed during the reductive conversion of 3-HP catalyzed by the purified protein (META1_2054). The protein was expressed on pET.32M.3C in the strain E. coli BL21 (DE3). Figure S6. Growth curve and 3-HP production in the strain YHP14. The gene pyk was amplified from M. extorquens AM1 genome, the amplified fragments were then cloned into pYM05 plasmid to construct pYM12 (PmxaF ::mcr-pyk). Plasmid pYM12 was then transformed into M. extorquens AM1 by electroporation to obtain the strain YHP14 (M. extorquens AM1/PmxaF ::mcr-pyk). [file 12934_2017_798_MOESM2_ESM.docx]

**Additional file 2**

**
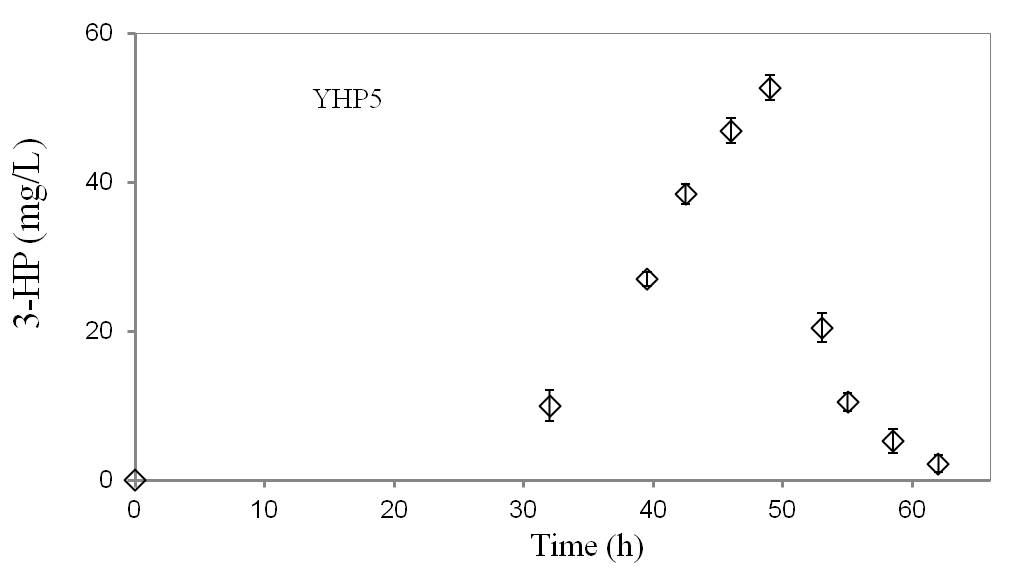
**

Figure S1 3-HP degradation at the transition from exponential phase to stationary phase of YHP5 (*M. extorquens* AM1/pJY80 (P*mxaF::mcr*)). Data was calculated from three independent biological replicates.


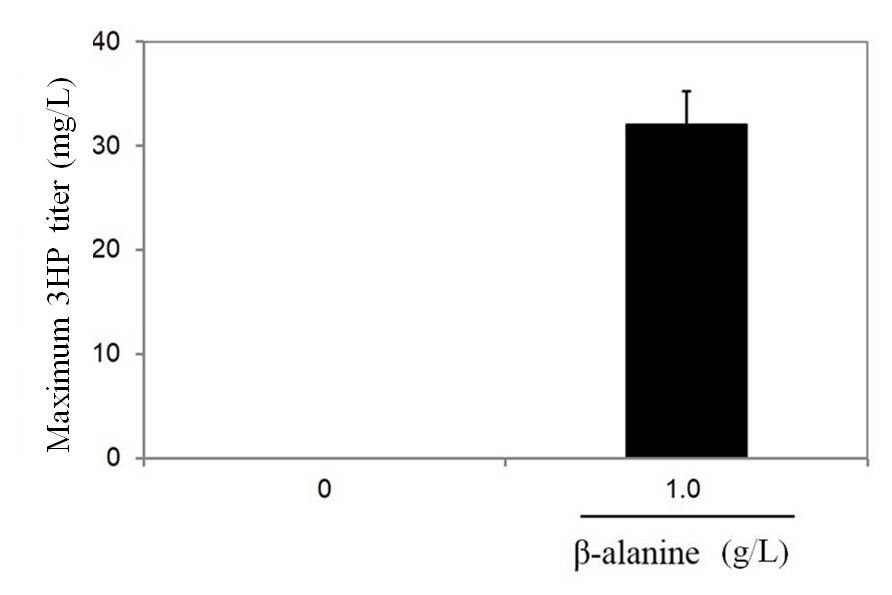


Figure S2 3-HP production in the YHP9 strain grown on methanol with the addition of β-alanine to the medium. X axis is the concentration (g/L) of β-alanine in the medium. YHP9 (BHBT5/pJY80 (P*_mxaF_* :: *yhxA*-*ydfG*)).


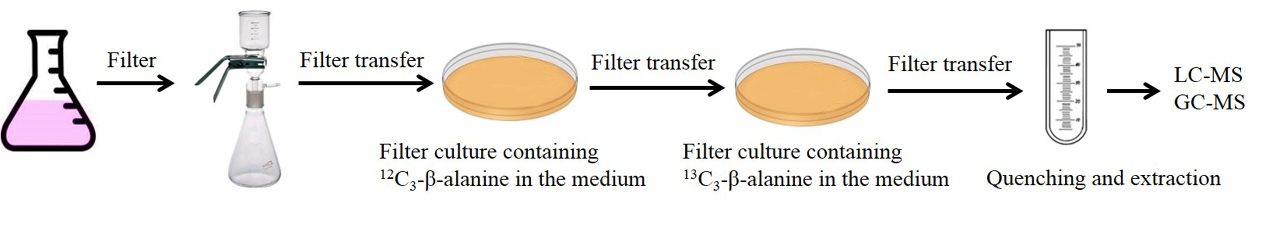


Figure S3 ^13^C-tracing experiment was carried out by switching from ^12^C-β-alanine to ^13^C-β-alanine in the YHP9 strain.


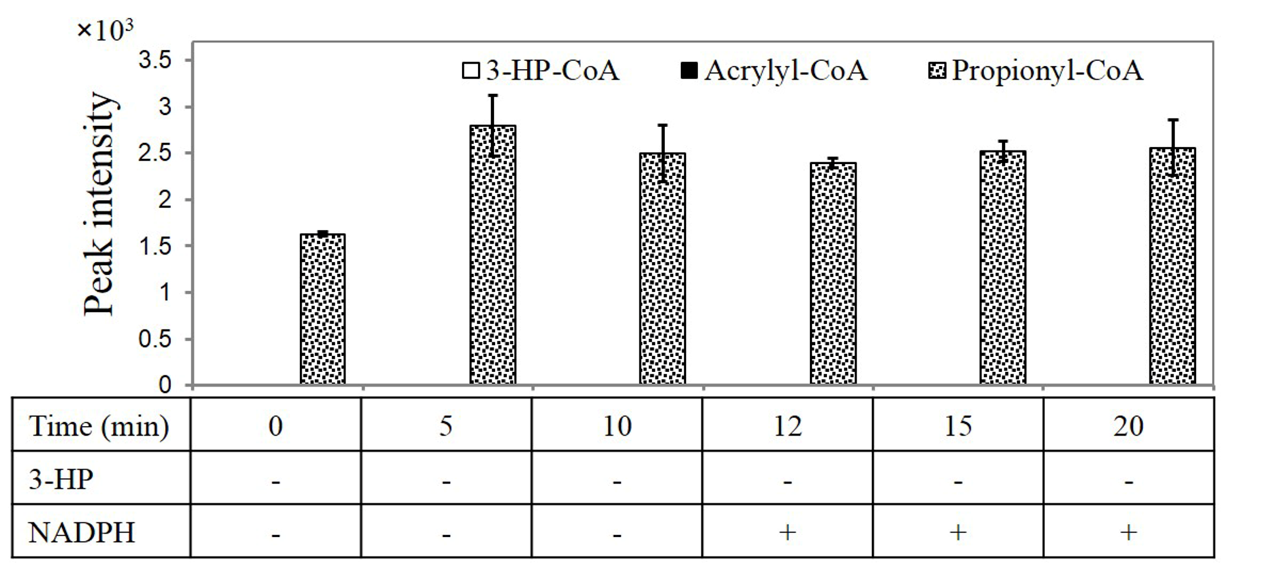


Figure S4 Control assay (i.e. no 3-HP addition) did not detect the accumulation of 3-HP-CoA and acrylyl-CoA by cell extracts of strain YHP8 in a time course. Shown are data for the reaction mixture for 10 min before the addition of NADPH and for 10 min after the addition of NADPH. YHP8 (BHBT5/pJY80).


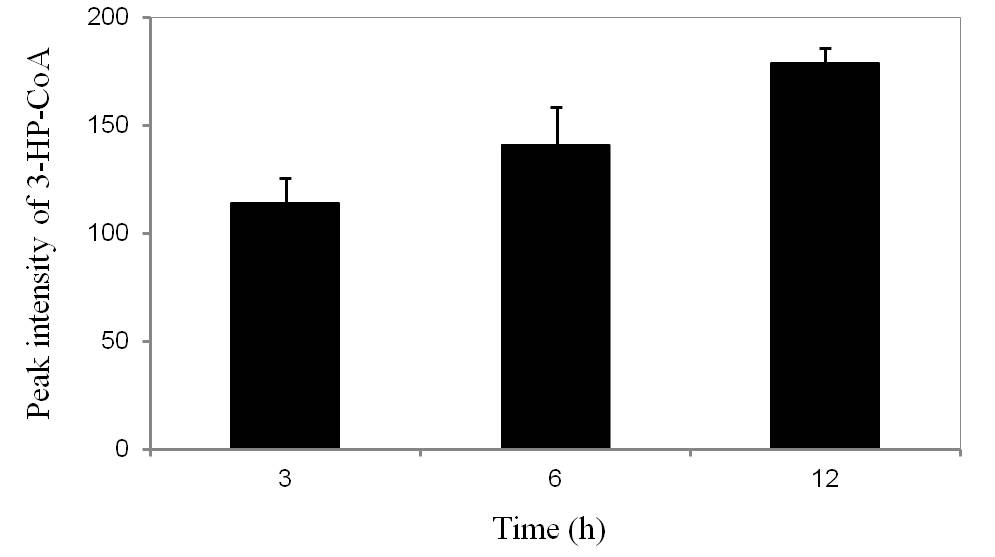


Figure S5 Analysis of 3-HP-CoA formed during the reductive conversion of 3-HP catalyzed by the purified protein (META1_2054). The protein was expressed on pET.32M.3C in the strain *E. coli* BL21 (DE3).


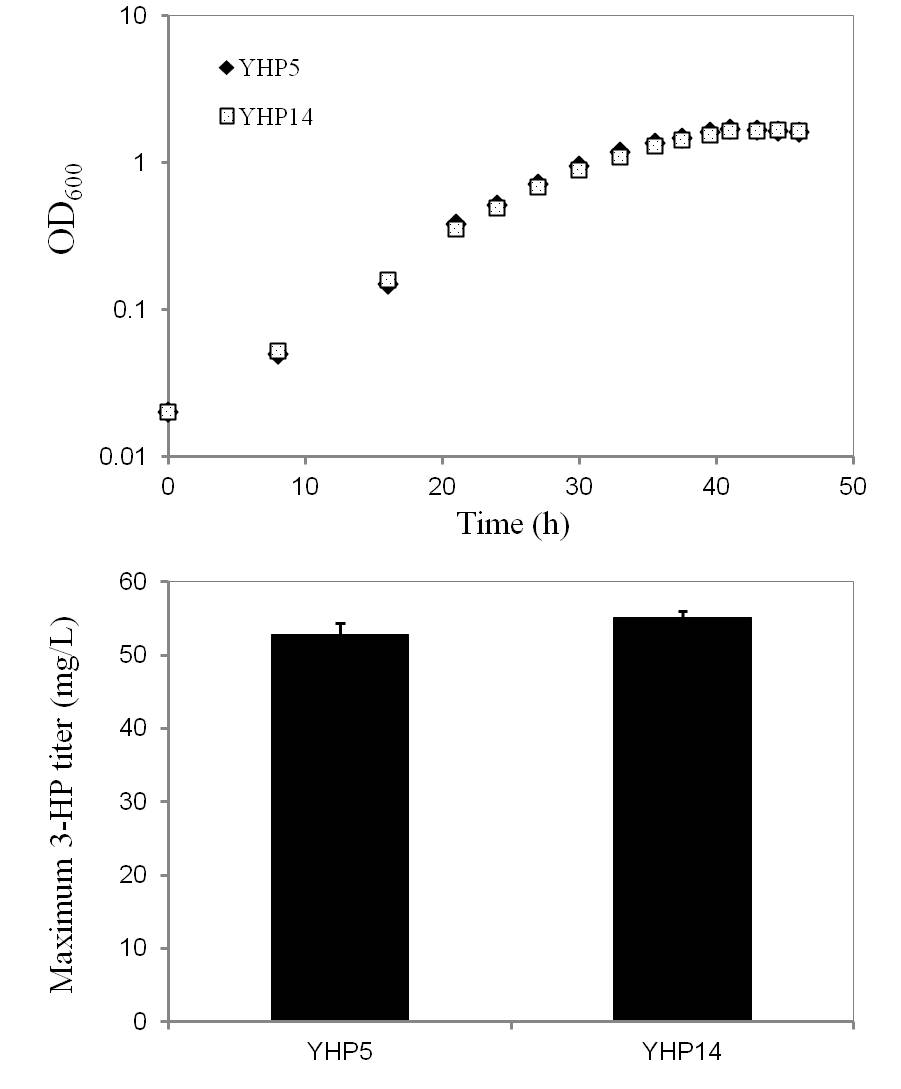


Figure S6 Growth curve and 3-HP production in the strain YHP14*.*

The gene *pyk* was amplified from *M. extorquens* AM1 genome, The amplified fragments were then cloned into pYM05 plasmid to construct pYM12 (P*mxaF::mcr-pyk)*. Plasmid pYM12 was then transformed into *M. extorquens* AM1 by electroporation to obtain the strain YHP14 (*M. extorquens* AM1/ P*mxaF::mcr-pyk*).
